# Percutaneous Spinal Needle–Assisted Suture Passing in Medial Meniscal Posterior Root Tear Repair

**DOI:** 10.1016/j.eats.2024.103245

**Published:** 2024-09-15

**Authors:** Ahmet Emin Okutan, Serkan Surucu, Kerim Öner, Lokman Kehribar

**Affiliations:** a Department of Orthopaedic Surgery, School of Medicine, Samsun University, Samsun, Turkey; b Department of Orthopaedics and Rehabilitation, School of Medicine, Yale University, New Haven, Connecticut, U.S.A.; c Department of Orthopaedic Surgery, School of Medicine, Karadeniz Technical University, Trabzon, Turkey; d Department of Orthopaedic Surgery, School of Medicine, Dokuz Eylül University, Izmir, Turkey

## Abstract

The medial meniscal posterior root (MMPR) is essential for absorbing hoop stress and preventing meniscal extrusion. MMPR tears lead to significant alterations in knee joint kinematics, increasing tibiofemoral contact pressure and accelerating knee osteoarthritis. Given the poor prognosis of untreated meniscal root tears, there has been growing interest in surgical repair techniques. The percutaneous spinal needle–assisted suture-passing technique offers a cost-effective, minimally invasive alternative that reduces iatrogenic damage to articular cartilage and eliminates the need for medial collateral ligament release compared with currently widely used suture passers. This technique allows for precise suture placement and minimizes meniscal injury during suture passing, providing a promising option for repair of the MMPR tear.

The medial meniscal posterior root (MMPR) plays a crucial role in absorbing hoop stress and preventing meniscal extrusion.^1^ After an MMPR tear (MMPRT), the altered knee joint kinematics lead to a significant increase in tibiofemoral contact pressure.^2^ This condition typically results in loss of hoop tension, loss of load-sharing ability, meniscal extrusion, unacceptable peak pressure on the cartilage, narrowing of the knee joint space, and rapidly progressing knee osteoarthritis.^2^^,^^3^ Owing to the poor prognosis of meniscal root tears, there has been growing interest in surgical repair of MMPRTs.^4^ The standard of care for meniscal root tears aims to restore the native function of the meniscal root and normalize tibiofemoral contact pressures.^5^

Transtibial pullout repair and suture anchor repair are the most used techniques to obtain anatomic fixation of the meniscal root.^6^^,^^7^ However, the achievement of appropriate suture configurations is challenging during surgical repair and requires the use of instruments such as suture passers within the small space provided by the knee with intact ligaments. Furthermore, suture-passing devices are likely to cause further iatrogenic damage to the cartilage. Therefore, we describe a percutaneous spinal needle–assisted suture-passing technique for MMPRT repair considering the disadvantages of existing techniques. The entire operational process does not require the use of costly consumables, resulting in increased cost-effectiveness.

## Surgical Technique

Our technique is presented in detail in Video 1.

### Indications and Contraindications

Indications for MMPRT repair include the presence of clinical and radiographic evidence of a radial tear located within 0 to 10 mm of the medial meniscal root attachment, which is confirmed during arthroscopy. Contraindications for meniscal root repair surgery include Kellgren-Lawrence grade 3 to 4 osteoarthritis, extensive grade 3 to 4 chondromalacia in the ipsilateral compartment, or patients deemed unable to meet with the postoperative rehabilitation protocol. Notably, moderate varus alignment of the lower extremity is usually addressed with a high tibial osteotomy performed concurrently with MMPRT repair.

### Patient Positioning and Anesthesia

Informed consent was obtained from the patient. The patient is positioned supine on the operating table. After the induction of regional anesthesia, a knee examination is conducted to check for any ligamentous instability and to assess the range of motion. A well-padded high-thigh tourniquet is then applied to the operative leg.

### MMPRT Repair Technique

**Fig 1:**
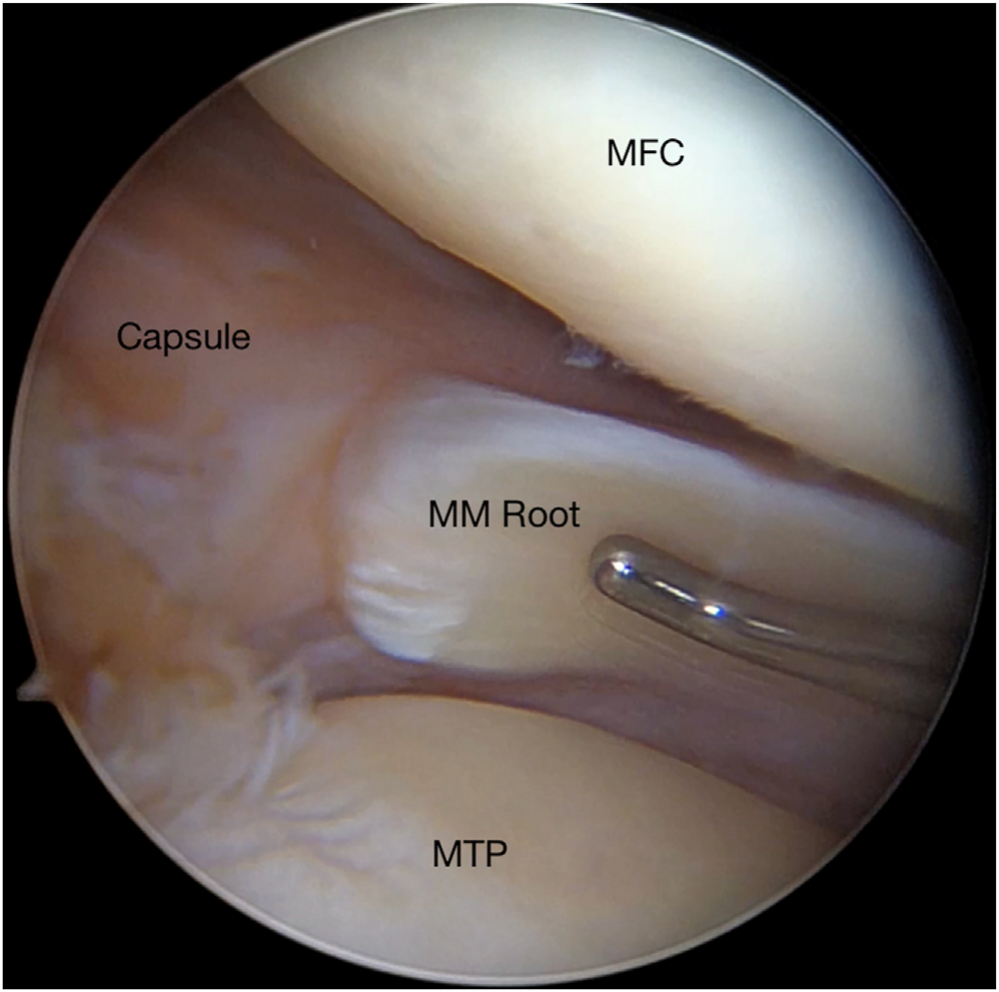
Arthroscopic view of right knee, identifying medial meniscal (MM) posterior root tear. (MFC, medial femoral condyle; MTP, medial tibial plateau.)

Standard anterolateral and anteromedial (AM) portals are created adjacent to the patellar tendon. The knee joint is insufflated with normal saline solution and visualized with a 30° arthroscopic camera (Stryker Endoscopy, San Jose, CA). An arthroscopic shaver (Stryker Endoscopy) is inserted into the knee, and any notable adhesions are removed. The medial compartment is examined under valgus stress. The medial meniscus is palpated with a probe, and the integrity of the whole meniscal body and posterior root is checked (Fig 1). After confirmation of the root tear, the insertion area of the MMPR is thoroughly prepared.

**Fig 2:**
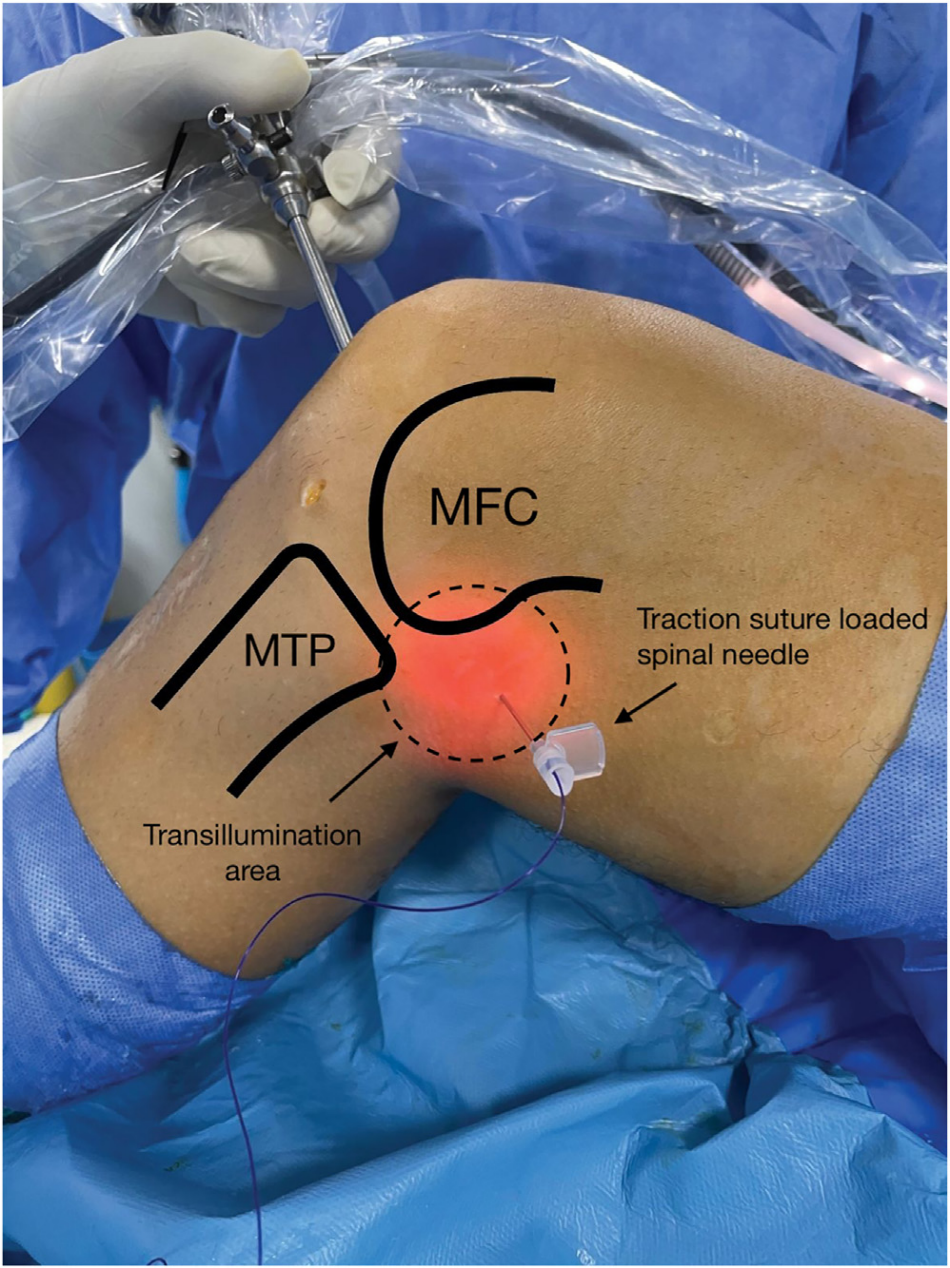
Medial view of right knee. The patient is in the supine position, with the knee in 90° of flexion. A traction suture–loaded spinal needle is introduced into the posteromedial area (circle) via a transillumination technique. (MFC, medial femoral condyle; MTP, medial tibial plateau.)

**Fig 3:**
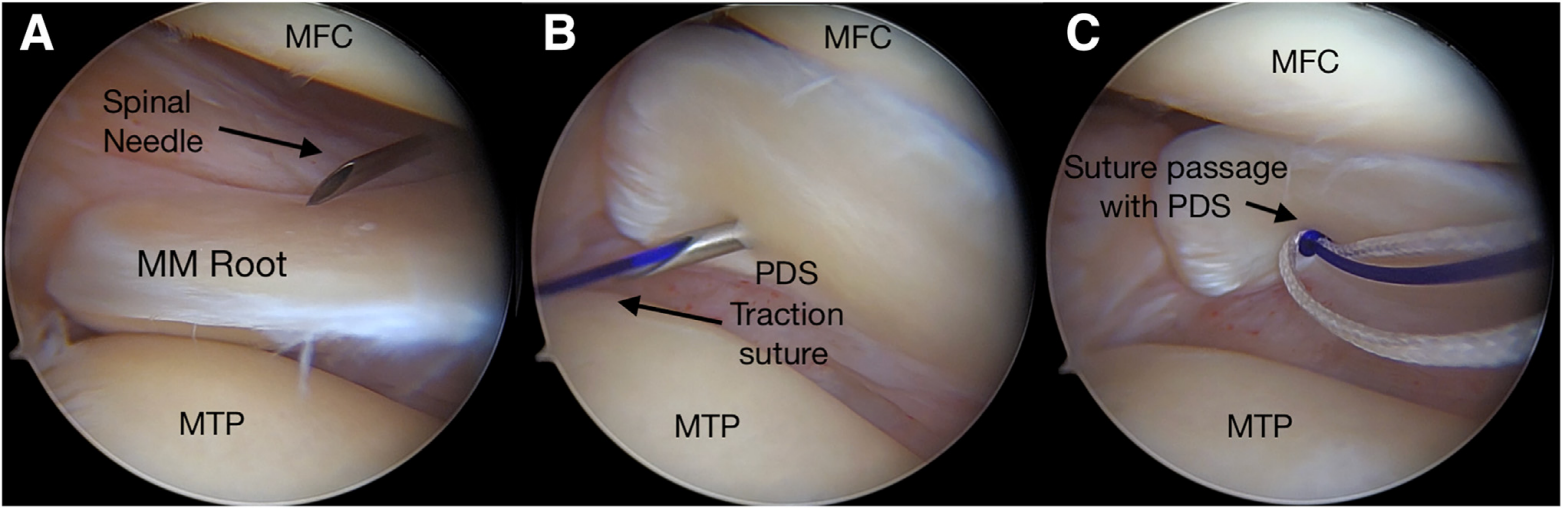
(A) The spinal needle is inserted percutaneously from the posteromedial aspect of the knee. (B) The meniscus is penetrated in the vertical direction, and the shuttle suture is pulled outside the anteromedial portal. (C) A high-strength nonabsorbable suture is passed into the meniscus through the shuttle suture (Right knee, supine position with knee under valgus stress in 30° of flexion). (MFC, medial femoral condyle; MM, medial meniscus; MTP, medial tibial plateau; PDS, polydioxanone.)

A 12-gauge spinal needle loaded with shuttle suture (PDS II; Ethicon, Somerville, NJ) is prepared. Then, the spinal needle is inserted percutaneously from the posteromedial aspect of the knee to penetrate the medial meniscus (Fig 2). This point is located at the soft spot between the medial head of the gastrocnemius, the medial collateral ligament, and the semimembranosus tendon, approximately 1.5 to 2 cm above the joint line. The spinal needle penetrates the medial meniscus from the femoral to tibial surface in the vertical direction, approximately 5 mm medial to its lateral edge for the medial meniscus, and the shuttle suture is pulled outside the AM portal using a grasper. No. 2.0 high-strength nonabsorbable suture is passed into the medial meniscus through the shuttle suture (Fig 3). The same procedure is repeated to insert additional suture into the meniscus. Then, all the suture limbs are pulled outside the AM portal using a suture retriever.

**Fig 4:**
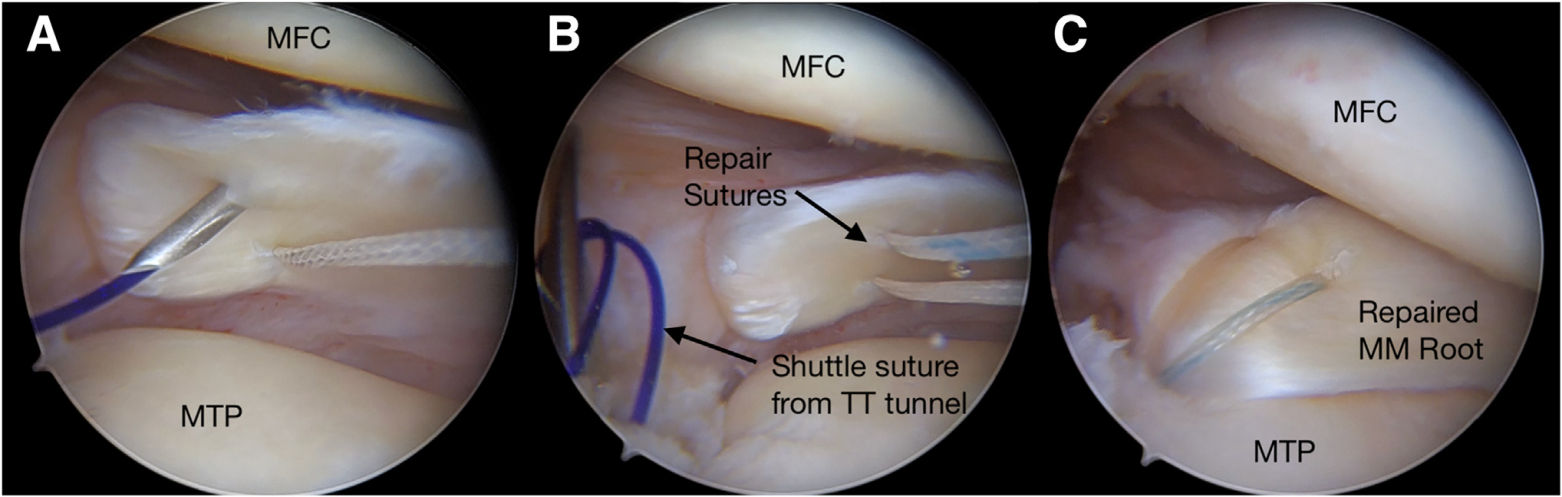
(A) The same procedure is repeated to insert additional high-strength nonabsorbable suture into the meniscus. (B) After transtibial tunnel (TT) creation, a looped passing suture is placed up the tunnel and retrieved out of the anteromedial portal. (C) Final view of medial meniscal (MM) root repair (Right knee, supine position with knee under valgus stress in 30° of flexion). (MFC, medial femoral condyle; MTP, medial tibial plateau.)

The location of the planned root repair on the tibial plateau is decorticated using a curved curette or a shaver. An initial incision for the transtibial tunnel is made just medial to the tibial tubercle about 5 cm distal to the joint line. A tibial tunnel guide is then used to ream the 2.4-mm tunnel at the location of the root attachment. The guide pin is visualized arthroscopically to verify correct tunnel placement, and the transtibial tunnel is created with a 3.5-mm reamer. Then, a looped PDS II passing suture is placed up the transtibial tunnel cannula, and the root sutures are shuttled down the tunnel. It is important to check that no synovial tissue is present between the sutures that have been pulled through the AM portal. All the sutures are retrieved through the AM portal together using the suture retriever to prevent soft-tissue bridge formation. Alternatively, a 5.5-mm cannula may be used through the AM portal to assist in suture management and prevent soft-tissue bridge formation. The MMPR sutures are retrieved through the joint and the transtibial tunnel using the looped polydioxanone suture as a shuttle. The reducibility and the correct position of the MMPR are checked (Fig 4). Finally, the root sutures are tied down using an SMC knot over a cortical fixation device (EndoButton; Smith & Nephew, Andover, MA) on the AM tibia, while the posterior root of the respective meniscus is visualized arthroscopically to confirm a secure repair.

### Postoperative Protocol

Physical therapy commences on the first day postoperatively, focusing on managing pain, reducing edema, and improving passive knee motion. The knee range of motion is restricted from 0° to 90° for the initial 2 weeks to protect the meniscal root repair, with gradual progression thereafter based on individual tolerance. Throughout the non–weight-bearing phase, which spans 6 weeks, the patient wears a knee immobilizer. Weight bearing is initiated at 6 weeks postoperatively. Avoidance of squatting and deep flexion for a minimum of 3 months after surgery is essential. Return to normal activities, including sports participation, is permitted after 6 months.

## Discussion

MMPRTs lead to meniscal extrusion and a loss of hoop stress and meniscal function, resulting in excessive stress on the articular cartilage of the knee and progression of knee osteoarthritis.^2^^,^^5^ Owing to the poor prognosis associated with meniscal root tears, there has been increasing interest in surgical repair of MMPRTs.^4^

Various surgical techniques and suture configurations have been described for MMPRT repair.^8^^,^^9^ It has been reported that the meniscus-suture interface, especially the suture cutout from the meniscus, contributes to displacement more significantly than any other aspect of the transtibial pull-out repair. In this context, several studies have investigated the biomechanical properties of specific meniscus-suture fixation techniques currently used in meniscal root repairs.^9^^,^^10^ They have concluded that reducing suture cutout at the meniscus-suture interface seems to be the most effective way to optimize the transtibial pullout repair technique.^9^^,^^11^ On the other hand, suture passing is an important component of root repair, and the diameter of the suture passer, which may also impact the structural properties of the meniscus, has not yet been evaluated. The spinal needle, being smaller than the smallest diameter of modern suture lassos or suture passers, creates the smallest possible hole in the meniscus. In addition, in a cadaveric study, Kim et al.^12^ reported the importance of the suture site to improving healing and facilitating the restoration of hoop tension for the MMPRT. The spinal needle–assisted suture-passing technique also enables a more precise determination of the suture-passing point.

Overall, the main feature of the described technique is that it is minimally invasive. There are many kinds of suture devices used for the meniscus, including suture passers and suture hooks. These devices can cause iatrogenic damage to cartilage tissue because they have large diameters. The spinal needle has a smaller diameter, resulting in less iatrogenic damage to chondral tissue. Moreover, it is used without requiring medial collateral ligament release. Additionally, the spinal needle is widely available and is less expensive than other suture-passing devices. We summarize the pearls and pitfalls of our technique in Table 1 and the advantages and disadvantages in Table 2. In conclusion, this method is considered safe, effective, convenient, and reproducible, thereby offering a promising option for MMPRT repair.

**Table 1:** Pearls and Pitfalls

Pearls
The spinal needle selected should be as small as possible to reduce iatrogenic injury to the meniscus. At the same time, the spinal needle must not be easy to bend and its diameter should be able to pass through the traction suture.
The position of the spinal needle should be adjusted percutaneously to effectively reach the meniscal root. If the puncture angle is not suitable, the spinal needle can be pulled out and the appropriate percutaneous puncture point can be selected again.
It is important to check that there are no soft-tissue bridges between the sutures. Before passing the sutures through the transtibial tunnel, all repair sutures and the passing suture should be pulled out through the anteromedial portal using a suture retriever.
Pitfalls
Repeated penetrations by the spinal needle can cause meniscal damage, potentially resulting in the sutures being pulled out.
The presence of soft-tissue bridges between the sutures may lead to suture tangling during passage through the tunnel.

**Table 2:** Advantages and Disadvantages

Advantages
The spinal needle tip, being smaller and sharper than the smallest diameter of modern suture lassos or suture passers, allows for increased penetration precision and creates the smallest possible hole in the meniscus.
Direct observation of the meniscal root enables a more precise determination of the suture-passing point.
The spinal needle has a smaller diameter than suture passers, resulting in less iatrogenic damage to chondral tissue.
The spinal needle–assisted technique can be applied without requiring medial collateral ligament release.
The spinal needle is widely available and inexpensive.
Disadvantages
Multiple percutaneous penetrations can cause injury to the saphenous neurovascular bundle.

## Disclosures

All authors (A.E.O., S.S., K.Ö., L.K.) declare that they have no known competing financial interests or personal relationships that could have appeared to influence the work reported in this paper.

## Supplementary Data

Video 1 Technique for percutaneous spinal needle–assisted suture passing in medial meniscal posterior root tear repair (Right knee, supine position with anterolateral viewing portal).
